# Circulating endothelial cell-derived extracellular vesicles mediate the acute phase response and sickness behaviour associated with CNS inflammation

**DOI:** 10.1038/s41598-017-09710-3

**Published:** 2017-08-29

**Authors:** Yvonne Couch, Naveed Akbar, Jay Roodselaar, Matthew C. Evans, Chris Gardiner, Ian Sargent, Ignacio A. Romero, Adrian Bristow, Alastair M. Buchan, Norman Haughey, Daniel C. Anthony

**Affiliations:** 1 Acute Stroke Programme, RDM-Investigative Medicine, University of Oxford, Oxford, UK; 2 Division of Cardiovascular Medicine, RDM, University of Oxford, Oxford, UK; 3 Department of Pharmacology, University of Oxford, Oxford, UK; 4 Haemostasis Research Unit, UCL, London, UK; 5 Department of Obstetrics and Gynaecology, University of Oxford, Oxford, UK; 6 Department of Life, Health and Chemical Sciences, The Open University, Milton Keynes, UK; 7 NIBSC, South Mimms, Potters Bar, UK; 8 Department of Neurology and Psychiatry, Johns Hopkins University, Baltimore, Maryland USA

## Abstract

Brain injury elicits a systemic acute-phase response (APR), which is responsible for co-ordinating the peripheral immunological response to injury. To date, the mechanisms responsible for signalling the presence of injury or disease to selectively activate responses in distant organs were unclear. Circulating endogenous extracellular vesicles (EVs) are increased after brain injury and have the potential to carry targeted injury signals around the body. Here, we examined the potential of EVs, isolated from rats after focal inflammatory brain lesions using IL-1β, to activate a systemic APR in recipient naïve rats, as well as the behavioural consequences of EV transfer. Focal brain lesions increased EV release, and, following isolation and transfer, the EVs were sequestered by the liver where they initiated an APR. Transfer of blood-borne EVs from brain-injured animals was also enough to suppress exploratory behaviours in recipient naïve animals. EVs derived from brain endothelial cell cultures treated with IL-1β also activated an APR and altered behaviour in recipient animals. These experiments reveal that inflammation-induced circulating EVs derived from endothelial cells are able to initiate the APR to brain injury and are sufficient to generate the associated sickness behaviours, and are the first demonstration that EVs are capable of modifying behavioural responses.

## Introduction

Any local damage to an organ, including the central nervous system (CNS), produces some degree of systemic inflammation by activating the acute-phase response (APR). Inter-organ communication by humoral factors such as cytokines is often suggested as a mechanism for the induction of the APR, and release of acute-phase proteins (APPs) into the circulation^1^. Following injury to the brain, the hepatic release of APPs occurs before there is significant evidence of an inflammatory response in the brain^2, 3^. TNF^4, 5^ and CXCL1^2, 6^ both fall into the APP category, and are rapidly synthesised by the liver as part of the APR in response to CNS injury.

The purpose of the APR is to promote the neutralization of pathogens by mobilizing the appropriate leukocyte populations, whilst simultaneously initiating repair processes. However, it is clear that over-activation of an APR can be detrimental^7^. The argument for humoral communication from the CNS to activate the APR post-injury is complicated by the lack of export sequences on cytokines produced in the CNS^8^. Indeed, experiments with recombinant cytokines, injected directly into the brain parenchyma or into the blood, suggest that the local release of free cytokine from the CNS into the circulation to target peripheral organs is not a major pathway^2, 9, 10^. Therefore, whilst the brain can mount a significant local inflammatory response to injury, the mechanisms by which this is communicated to the periphery remain unclear.

Extracellular vesicles (EVs) are membrane-enclosed vesicles comprising larger (100nm-1µm) microvesicles (MV), formed by the outward budding and fission of the plasma membrane, and exosomes, which are smaller (<200 nm) and formed by the endocytic invagination of endosomal membranes and stored in multivesicular bodies (MVB)^11^. Platelet-derived EVs are produced in large numbers, but EVs have also been shown to be produced by leukocytes^12^, endothelial cells^13^, neutrophils^14^ and macrophages^15^.

Whilst the presence of EVs in the blood is a normal physiological phenomenon, many pathologies have been found to be associated with considerable increases in circulating EVs, including inflammatory and autoimmune diseases, atherosclerosis, and malignancies^16, 17^. Brain injury and disease is no exception, however the role of circulating EVs in CNS pathology has remained unclear^18^. Endothelial cells are placed conveniently at the junction between the CNS and the circulation and - playing a key role in barrier function - seem an ideal candidate for EV-mediated communication of CNS injury^19^. For example, in acute ischemic stroke, endothelial EVs have been shown to be positively correlated with lesion volume and stroke outcome^20^, but, once again, it is not clear whether they contribute to the pathogenesis of stroke, or whether they are simply produced as a consequence of it. Ischaemic stroke is known to generate a significant systemic APR^21^, and as such endothelial EVs may have a role in co-ordinating the inter-organ inflammatory signalling pathways responsible for this, and thus provide a particularly compelling route for CNS-liver communication. Indeed, recent studies have shown that endothelial EVs are capable of transferring molecules to leukocytes during CNS inflammation^22^, however, as so little is currently known about the role of CNS EVs *per se*, studies in a basic model of CNS inflammation are key to future understanding of their role in disease.

Against this background, we sought to study the function of circulating EVs following the induction of a focal interleukin-1β (IL-1β)-induced inflammatory lesion within the brain parenchyma, followed by blood fractionation to transfer EVs between animals to test the hypothesis that EVs elicited after injury are a key mediator of CNS-liver communication. Compared to other acute brain injury models, the IL-1β challenge generates a focal inflammatory response with stereotypical sickness behaviours^5^ in the absence of blood-brain barrier breakdown or acute cell death in adult animals^23^. The single-bolus injection of IL-1β employed here induces *de novo* synthesis of endogenous IL-1β^24^ by microglia and endothelial cell activation^25, 26^, as well as continued leukocyte recruitment for up to seven days^24^. We used this approach to investigate whether the transfer of the EV fraction alone, from the blood of an IL-1β-challenged animal to a naïve animal, would result in an increase in hepatic APPs and the subsequent associated sickness behaviours^5, 10, 27^. Confirmation that EVs from brain endothelial cells alone could account for the molecular and behavioural changes observed was obtained from cultured brain vascular endothelial cells exposed to an IL-1β challenge. The results described here show that not only are EVs able to initiate an APR, but they are also able to activate the sickness behaviours associated with injury or infection, thus showing that more complex behavioural phenotypes can be transferred simply by transferring only the EV fraction of the blood. The recognition that EVs are not simply passive biomarkers of disease, but a targeted inter-organ communication system provides a whole new avenue for the development of future therapies treating inflammatory disease in the brain and, most likely, peripheral inflammatory pathologies.

## Results

### Number of circulating EVs increases in animals after central IL-1β injection

**Figure 1:**
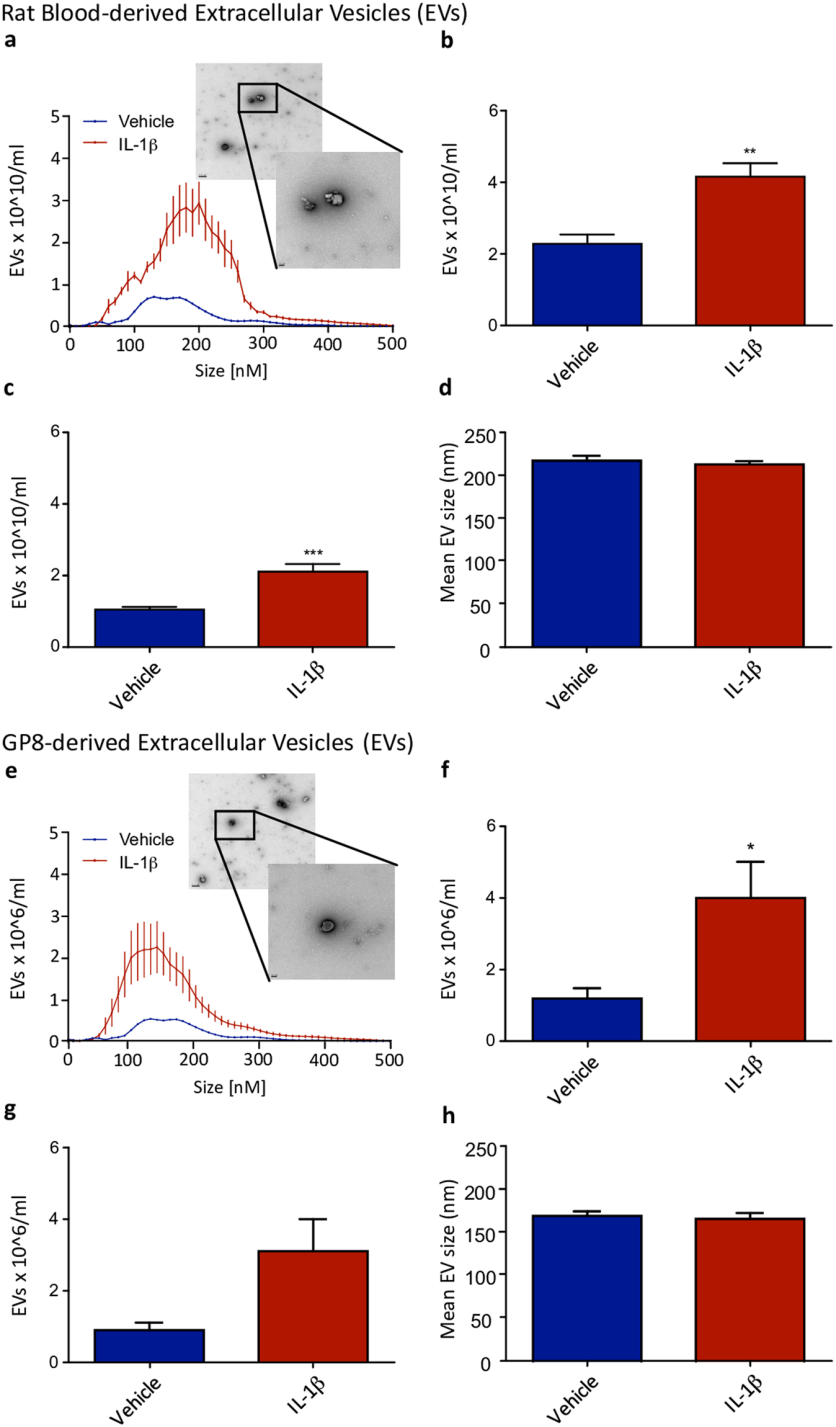
Extracellular vesicle (EV) release *in vivo* and *in vitro* after an IL-1β challenge. Animals received a single injection of IL-1β (10ng in 1 µl into CNS parenchyma within the striatum) or vehicle (saline) and blood was taken at 2 hours and analysed for EV number and size using Nanosight Tracking Analysis. Distribution in vehicle and IL-1β treated animals demonstrated a mixed population of vesicles (**a** - graph; EM inset). This was quantified as EVs × 10^10^/ml by NTA for vesicles <200 nm (**b**) as well as for total vesicles (**c**). Average size of the EV population in nm was also determined (**d**). Brain vascular endothelial cells (GP-8) were treated with IL-1β (10ng/ml) for 2 hours and supernatant was harvested and analysed for EV size and number using NTA. Distribution in vehicle and IL-1β treated cells demonstrated a mixed population of vesicles (**e** - graph; EM inset). This was quantified as EVs × 10^6^/ml by NTA for vesicles <200 nm (**f**), as well as for total vesicles (**g**). Average size of the EV population in nm was also determined (**h**). Data are mean ± SEM, n = 6, *p < 0.05; **p < 0.01 and ***p < 0.001. Scale bars on micrographs show 0.2 µm and 50 nm (inset).

EV release occurs under normal physiological conditions, and has been shown to increase in inflammatory pathologies^28^. To determine whether CNS inflammation elicited an increase in EVs, we employed a generic model of CNS inflammation^2, 24, 29, 30^. Animals were allowed to survive for 2 hours after the stereotactic microinjection of IL-1β (10ng) into the brain (striatum), which has been previously shown to generate a significant NFkB-dependent acute phase response in the liver^2, 31^ in the absence of any increase in IL-1β in the plasma (supplementary Fig. S1) We used nanoparticle tracking analysis (NTA) to determine the size and number of EVs in the blood after the injection. Compared to vehicle, IL-1β increased the number of circulating EVs (Fig. 1a,b,c). In particular, there was a peak in particles of approximately 200 nm (Fig. 1a), with a significant treatment:size interaction (F_50,510_ = 9.79, p < 0.0001; Fig. 1a). Despite this interaction, there was no difference in size of EVs overall (Fig. 1d), and the increase in EV number was seen for both small (<200 nm; Fig. 1b) and total EV populations (Fig. 1c), suggesting that size distribution was largely unaltered by IL-1β treatment (Student’s t-test p < 0.0001 and p < 0.001, respectively).

We next sought a candidate cell type that could be responsible for the increased production of EVs, and for this we used a rat endothelial cell culture model, because of the position of the endothelial cells, poised as they are at the junction of the CNS and periphery, and their known response to pro-inflammatory signals^24, 32, 33^. Our EV population from our IL-1β injected animals was shown to be enriched for CD31 out of proportion to CD41 expression (supplementary Fig. S2), supporting an endothelial cell origin. We therefore challenged rat brain vascular endothelial cells (GP-8 rat brain-derived) with IL-1β (1ng/ml) for 2 hours, so that the time frame was comparable to the *in vivo* experiments. IL-1β significantly increased EV shedding compared to controls (Fig. 1e, f, g), with a peak in particles of 200 nm and a significant interaction between treatment and size (F_50,204_ = 4.16, p < 0.0001). In a similar manner to the *in vivo* model, we detected no overall change in size of the EVs (Fig. 1h), but an increase in smaller EVs (<200 nm; Student’s t-test p < 0.05; Fig. 1f). Thus brain endothelial cells are clearly capable of shedding EVs in response to the presence of IL-1β. It should be noted that no IL-1β could be detected in the EV fraction or in the ultracentrifuged, pooled EV-free fraction (supplementary Fig. S1).

### Transfer of EV fraction after inflammatory challenge results in a hepatic inflammatory response

**Figure 2:**
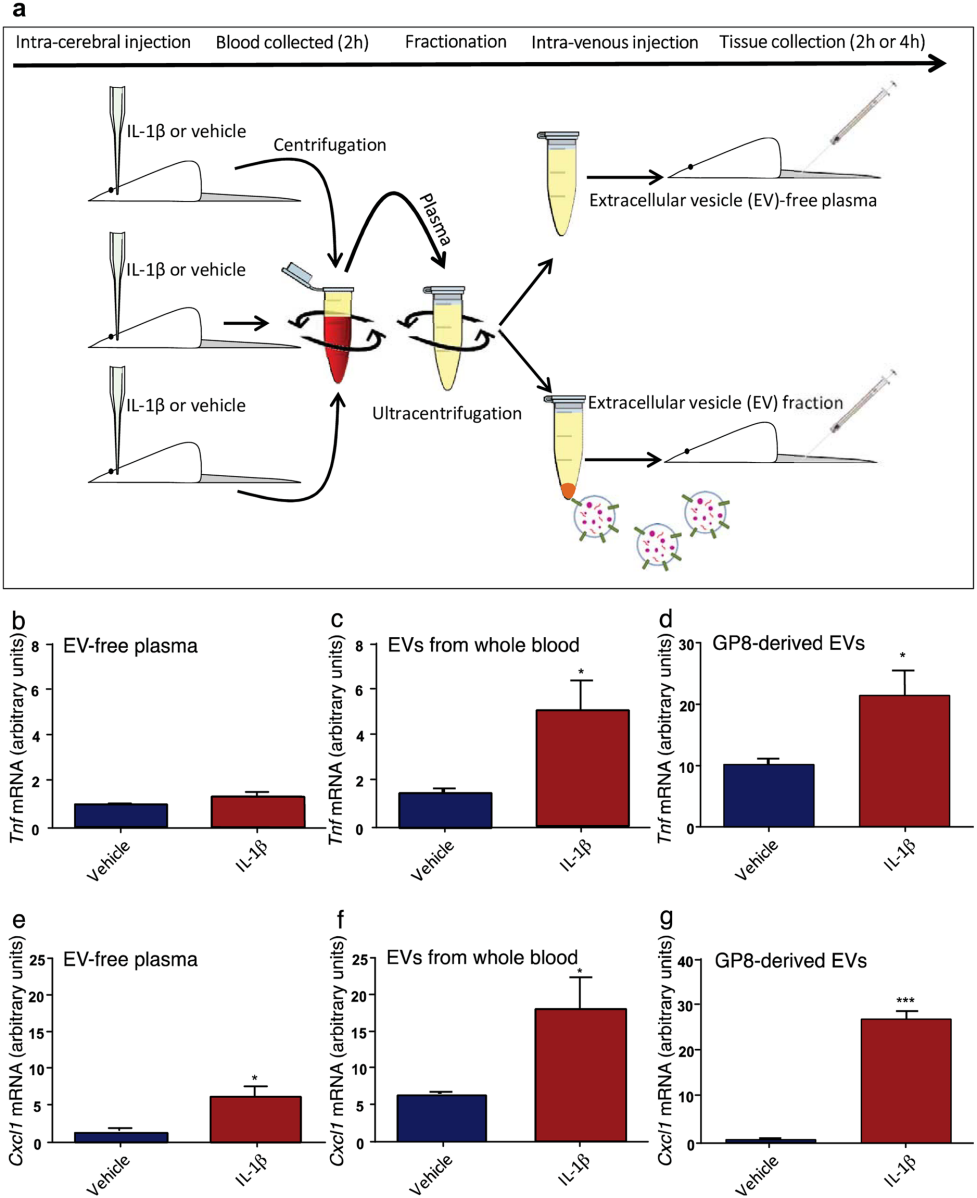
Schematic overview of *in vivo* experiments (**a**). Animals received a single intracranial injection of IL-1β or vehicle (saline) and blood was collected and processed for EV isloation. Platelet free plasma was separated using centrifugation and EVs further separated by ultracentrifugation. Samples from 3 ‘donor’ animals were pooled and administered to each ‘recipient’ animal. The hepatic response to EV transfer of *in vivo* and *in vitro-*derived EVs (**b–e**): EVs or EV-free supernatant were transferred from donor animals challenged with IL-1β or saline into naive recipient animals and the hepatic response was studied after 4 hours. Specifically, expression of TNF mRNA in naïve animals receiving EV-free supernatant (**b**); EVs isolated from whole blood **(c**) or EVs from GP-8 cell supernatant (**d**). CXCL-1 mRNA in animals receiving EV-free supernatant (**e**); EVs isolated from whole blood (**f**) or EVs from GP-8 cell supernatant (**g**). All qPCR data are normalized to the expression of the housekeeping gene GAPDH and then further normalized to control animals. Data are mean ± SEM, n = 6, *p < 0.05 and ***p < 0.001.

Previous work from our group has shown that a pro-inflammatory insult to the CNS causes an early hepatic response, with induction of NFκB-regulated genes, including pro-inflammatory cytokines and chemokines^9, 34^, leading to neutrophil recruitment. In order to find out whether the circulating EVs released from the CNS after an IL-1β challenge were capable of inducing a hepatic inflammatory response, EV-free plasma supernatant and EV fractions were pooled and transferred from donor animals, injected with either IL-1β or saline, into naïve recipient animals (Fig. 2a).

Using quantitative PCR we showed that EV-free supernatant from the plasma of IL-1β treated animals does not induce TNF mRNA expression in the liver (Fig. 2b) but the EV fraction, isolated from EV-free supernatant and introduced into naïve animals, induced a 5-fold increase in TNF mRNA expression compared to vehicle controls (Student’s t-test; p < 0.05; Fig. 2c). Plasma-derived EV-free supernatant transfer induced a small increase in CXCL-1 mRNA in IL-1β injected animals compared with EV-free supernatant from vehicle treated animals (Student’s t-test p < 0.05; Fig. 2e). The transfer of the EV fraction from IL-1β challenged animals to naïve animals resulted in a large increase in CXCL-1 expression (p < 0.05; Fig. 2f). As was the case for the *in vivo* transfer, EVs derived from IL-1β treated cells significantly increased production of TNF (Student’s t-test p < 0.05; Fig. 2d) and CXCL-1 (p < 0.001; Fig. 2g) compared with injection of EVs from saline treated cells. Thus, not only is the EV fraction sufficient for transfer of a pro-inflammatory state in the liver, but EVs isolated specifically from rat brain vascular endothelial cells are sufficient for this purpose.

**Figure 3:**
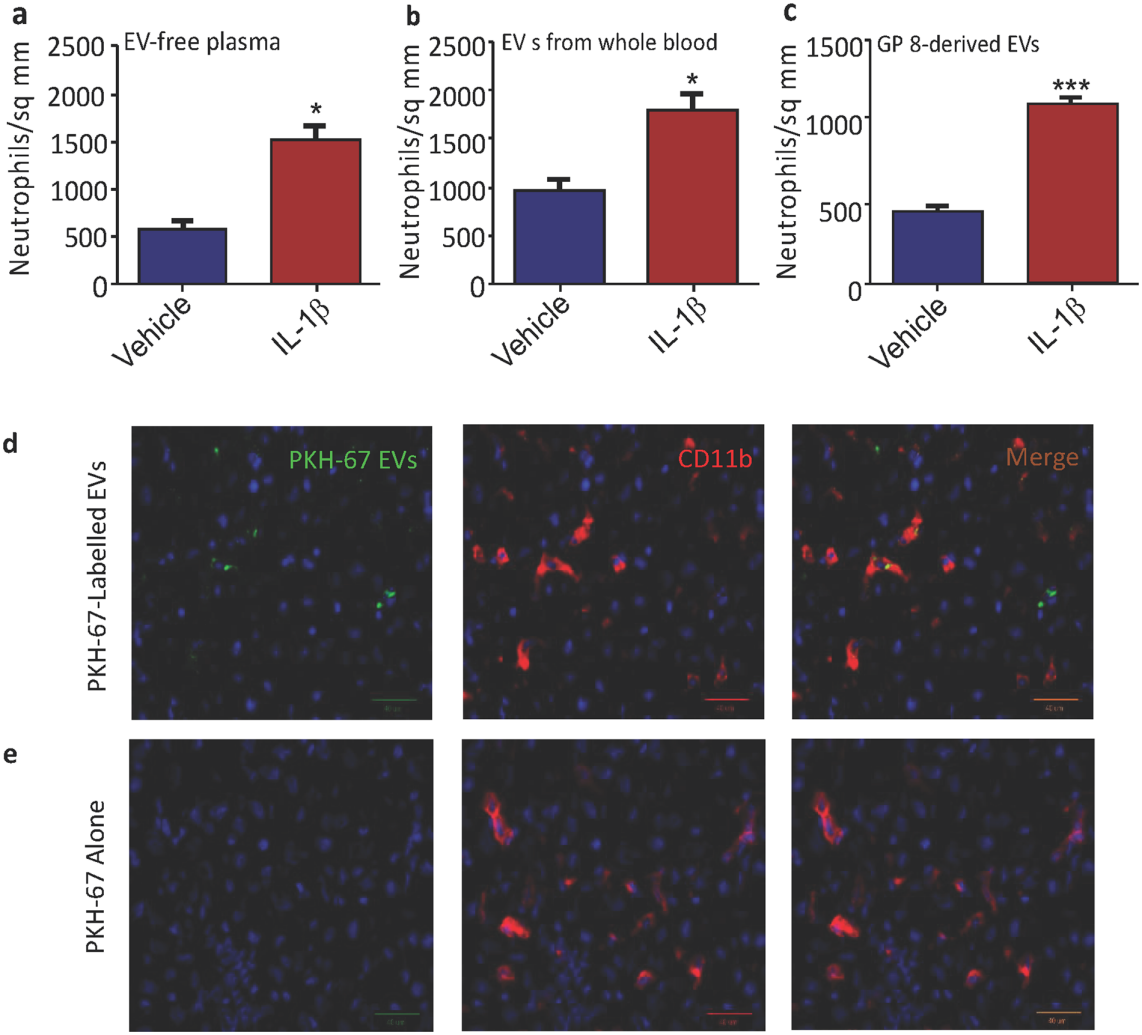
Hepatic neutrophil numbers were counted in animals receiving EV-free supernatant (**a**); EVs isolated from whole blood (**b**) or EVs from GP-8 cell supernatant (**c**). Representative microscopy of PKH-67-labelled EVs (green) introduced into naïve animals tracked to the liver (**d**) where they were found largely in CD11b-positive (red) Kupffer cell populations. An administration of PKH-67 intravenously does not result in any staining (**e**). Scale bars on micrographs represent 40 µm.

Finally, given that one of the consequences of increased hepatic chemokine expression is neutrophil chemotaxis, we examined the livers of the recipient rats using light microscopy using an in-house generated anti-neutrophil antibody. For *in vivo*-derived EVs transferred to recipient rats, there were significantly more neutrophils in rats given EVs compared with vehicle (p < 0.0001; Fig. 3b), a pattern that was recapitulated with rats given *in vitro*-generated EVs treated with IL-1β compared with vehicle (p < 0.0001; Fig. 3c). However, in addition to this, there were also increased numbers of neutrophils in the livers of rats given EV-free plasma compared with saline (p < 0.0001; Fig. 3a), suggesting that both the EVs, *and* some humoral substance, are capable of causing hepatic neutrophil chemotaxis.

### EVs generated from IL-1β-treated rats track to hepatic Kupffer cells

**Figure 4:**
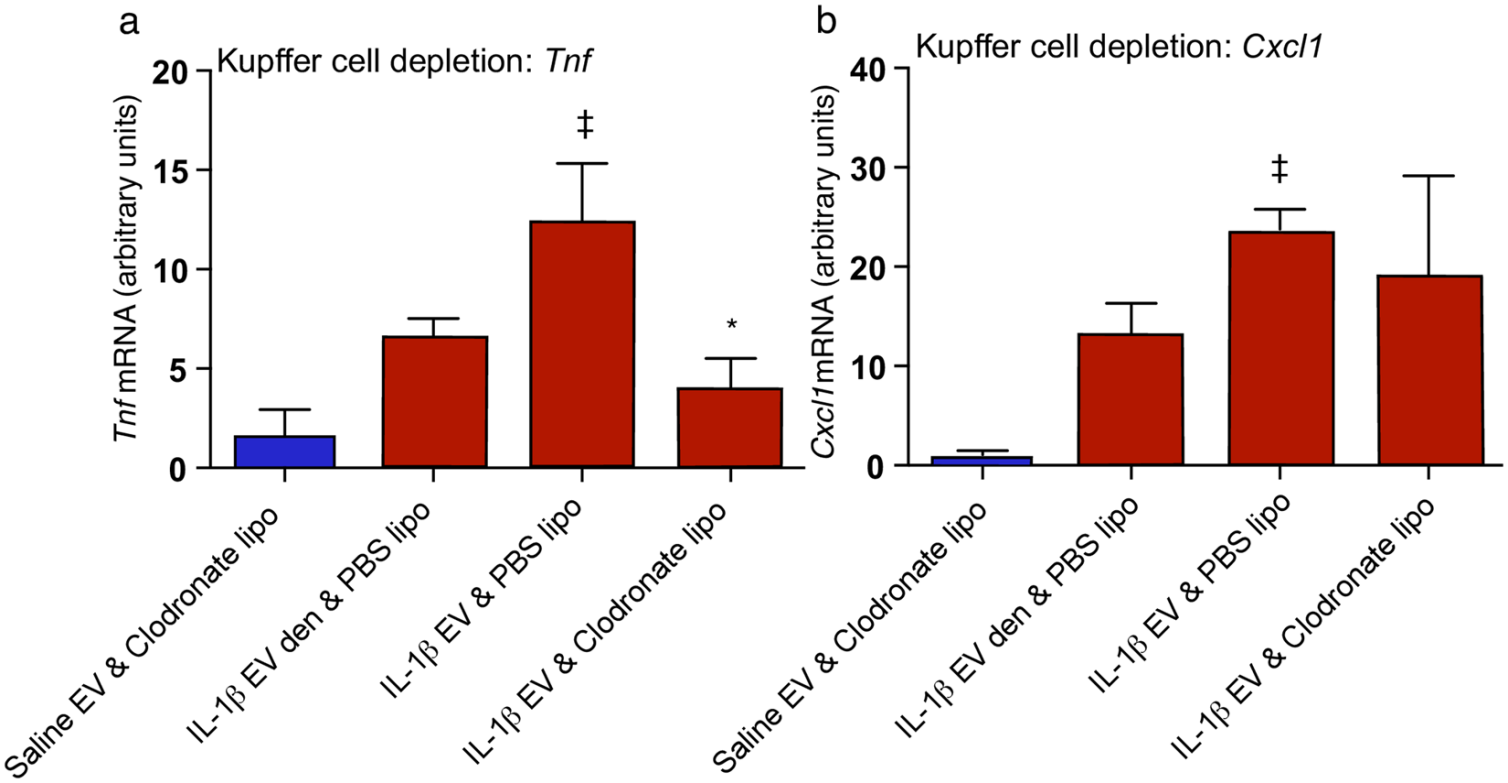
The hepatic response to EV transfer of *in vitro-*derived EVs in clodronate-treated animals. (**a**) TNF mRNA expression in the liver of animals receiving EVs isolated from IL-1β treated GP-8 cell supernatant in the presence or absence of clodronate-filled liposomes (lipo) or denatured EVs (EV den). (**b**) CXCL-1 mRNA expression in the liver of animals receiving EVs isolated from IL-1β treated GP-8 cell supernatant in the presence or absence of clodronate-filled liposomes. All qPCR data are normalized to the expression of the housekeeping gene GAPDH and then further normalized to control animals. Data are mean ± SEM, n = 4; ^‡^compared to saline/liposome treated animals; *compared to IL-1β treated animals.

We next wished to examine whether EVs injected systemically in recipient rats localized to the liver. To achieve this we fluorescently labelled extracted EVs with the membrane dye PKH-67, before injecting them into a cohort of recipient rats, and then analysed specific organs using fluorescent microscopy. Whilst fluorescence was largely absent from other organs such as the spleen, lung and heart (supplementary Fig. S4), it was detectable in the liver (Fig. 3d). We suspected that the Kupffer cell population would be responsible for this uptake through phagocytosis, and co-labelling with the macrophage marker CD11b showed good co-localization of fluorescence signal (Fig. 3d), in support of this hypothesis. Here, it should be noted that free dye injected into animals does not result in labelling (Fig. 3e). Further evidence in support of this hypothesis was found in animals pre-treated with clodronate liposomes. These deplete peripheral macrophage populations, including liver Kupffer cells. In animals treated with clodronate, an i.v. injection of IL-1β EVs generated from GP-8 cells failed to elicit an inflammatory response to the same degree as IL-1β EVs and empty liposomes (Fig. 4). Indeed, TNF expression was significantly increased after animals received EVs from IL-1β treated endothelial cells when compared to saline/clodronate treated animals (Student’s t-test p < 0.01; Fig. 4a), and this increase was reduced in animals receiving EVs/clodronate (p < 0.05; Fig. 4a). CXCL-1 expression showed a similar pattern, with a significant increase in EV treated animals when compared to saline/clodronate animals (p < 0.05; Fig. 4b) which was slightly decreased in animals receiving clodronate prior to EVs.

### Extracellular vesicles are capable of inducing sickness-behaviour in recipient animals

Previous studies from our group have shown that inflammatory insults to the brain result in “depression-like” sickness behaviours, that are at least partly dependent on cytokine and chemokine expression in the liver^5, 10, 35^. Thus, given that we have shown that transfer of EVs from IL-1β-treated rats and rat-derived endothelial cells migrate to the liver, thereby inducing a hepatic acute phase response, it seemed intuitive that this might also result in the transfer of sickness behaviours to recipient rats.

**Figure 5:**
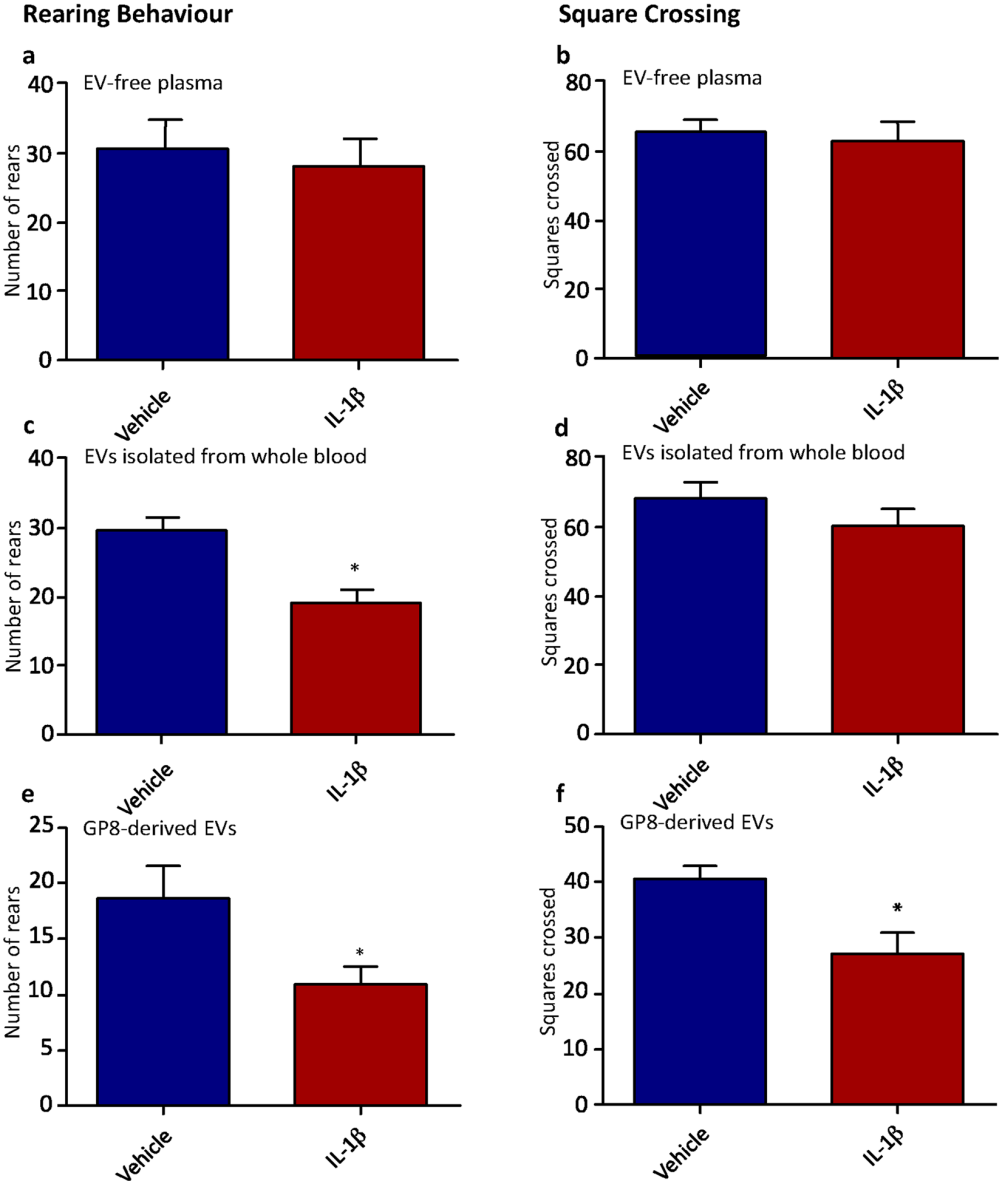
The behavioural response to IL-1β-stimulated, *in vivo*-derived circulating EVs and *in vitro-*derived brain endothelial EVs. Number of rears and number of squares crossed in the open field are shown for rats given *in vivo*-derived EVs or EV-free plamsa (**a**–**d**), and also for EVs from IL-1β and saline-treated endothelial cells (**e**,**f**). Data are mean ± SEM, n = 6, *p < 0.05 and **p < 0.01.

We used the open field paradigm to measure the number of rears performed by each animal and the number of squares crossed, a well-validated measure of exploratory behaviour that shows significant changes in models of sickness behaviour^27, 35, 36^. For the *in vivo* transfer of EVs, recipient rats showed significantly fewer rears on the open field (Student’s t-test; p < 0.05; Fig. 5a,b), and a trend to fewer number of squares crossed (Fig. 5c,d). We found a similar result for recipient rats injected with *in vitro*-generated EVs, with significantly fewer rears (Student’s t-test; p < 0.05; Fig. 5e) and squares crossed on the open field (p < 0.05; Fig. 5f). Taken together this is the first published report of a behavioural phenotype resulting from the transfer of EVs from “sick” animals/cells to healthy recipients, and highlights how systemic and wide-ranging the role is of EVs.

## Discussion

Injury to the CNS normally results in the induction of a systemic acute phase response (APR), which is capable of exacerbating the central injury. Abrogation of this response could prove useful in acute injuries, such as traumatic brain injury or stroke. Until now the signals responsible for the activation of the APR after brain injury were not known, but speculated to be humoral or neural. Our study shows that brain endothelial cell-derived extracellular vesicles (EVs) are sufficient to mediate not only brain-liver communication after injury, but also inflammation-induced sickness behaviour. The striking observation that the circulating EV fraction is sufficient to elicit an APR when they are transferred from a brain-injured animal to a naïve animal, reveals that non-neural, non-humoral communication provides an explanation for the selective activation of the peripheral APR, and provides a new target for therapeutic intervention in acute brain injury. In addition, discovery that the EV fraction from an injured animal is sufficient to elicit sickness behaviours in the recipient was wholly unexpected and as such introduces an entirely new concept of EV mediated behaviour to the psychoneuroimmunology field.

In this study, we employed a well-characterised model of CNS inflammation to explore brain-liver communication^37^, and more specifically the role of endothelial cell-derived EVs in this pathway. The microinjection of IL-1β into the brain parenchyma generates a focal inflammatory lesion, and there is known to be no sensible leakage of cytokine into the circulation from the brain^2, 9^. As such, this minimally invasive model lends itself to the study of brain-to-peripheral immune system responses. The concentration of IL-1 in the present study is equivalent to that generated after a haemorrhagic stroke^38^. However, we have previously shown that these IL-1β-induced focal brain inflammatory lesions rapidly induce hepatic NFkB-dependent cytokine and chemokine expression^31^, causing mobilisation of leukocytes from the marrow to the blood and then into the injured brain^34^, as well as chemotaxis to the liver^2^. This pathway has been shown to be reliant on hepatic chemokine production, which is also a feature of chronic immune-mediated CNS disease where EVs have been shown to be elevated^10^.

Broadly, inflammation is known to cause an increase in EV production from a number of cell types. The majority of studies focus on platelet-derived EVs but they have also been shown to be produced by leukocytes^39^, endothelial cells^13^, astrocytes^40, ^ neutrophils^14^ and macrophages^15^. Here, we have demonstrated that there is an increase in EV release from both the injured CNS, and from brain endothelial cells in culture after IL-1β stimulation. Endothelial cells are likely to be an important mediator of inter-organ signalling *in vivo*, owing to their unique position at the interface of CNS and the blood. Vascular endothelial cells are known to express receptors for IL-1β and upregulate markers of inflammation in response to an IL-1β challenge^24, 32, 33^, as well as to shed EVs under pathological conditions^19^. Vascular cells are exclusively placed at the CNS-immune junction. Given the findings in this study of elevated TNF and CXCL-1 production after administration of EV-fraction from inflammatory challenged animals or cells, it seems that at least one major mechanism underlying this CNS-liver inflammatory signalling is the production of vascular endothelial EVs. This theory is supported by the recent work of Paul and colleagues, who have demonstrated that endothelial EVs transfer claudin-5 to leukocytes during episodes of CNS inflammation^22^. Whilst our data demonstrate that EVs are shed from endothelial cells in response to an IL-1β challenge, it is probable that other cell populations within the CNS may also release EVs that contribute to the cross-talk between the injured brain and the peripheral immune system. For example, astrocytes generate large membrane-enclosed vesicles that are able to cross membranes^40, 41^, and it is likely these cells may act in concert to co-ordinate the response to injury.

Our data also demonstrate that isolated EV fractions from both IL-1β treated animals, and from IL-1β challenged cells, are capable of inducing neutrophil recruitment, an effect that was also found for EV-free plasma which, taken together, suggests that whilst there is also some humoral molecule capable of signalling hepatic neutrophil recruitment, the EVs themselves are sufficient for this process. This finding is consistent with the cytokine and chemokine expression, in that CXCL-1 is produced at an elevated level after administration of both EV-free plasma and the EV fraction from rats/cells treated with IL-1β, with the latter having an even higher expression. CXCL-1 is an important chemokine involved in neutrophil transport^42^, so we would predict that CXCL-1 expression and neutrophil recruitment go together, as, indeed, they do here. Interestingly, a recent study by Dickens *et al*.^40^, has demonstrated that EVs derived from stimulated astrocytes do not produce significant hepatic expression of CXCL-1 when they are injected into naïve recipients, but they do induce hepatic TNF. This data suggests that EVs from distinct cell populations may carry their own unique injury signals to the periphery, and that the resultant characteristics of the peripheral immune response is dependent upon the combined effects of the total EV population. This phenomenon may enable fine tuning of the peripheral inflammatory response to CNS injury to ensure that the most appropriate leukocyte populations are mobilized.

Irrespective of cell of origin, after biogenesis in the CNS, blood-born EVs can be captured by any number of inflammatory cells throughout the body. However, our previous data has demonstrated that there is a significant hepatic response to CNS injury^2^. When we administered fluorescent EVs intravenously we found them mainly in the liver. We suspected that the EVs were being removed by hepatic Kupffer cells, and our immunofluorescent imaging of CD11b + macrophages and labelled EVs, show overlap of fluorescent signal, indicating that the sequestration was, predominantly, if not completely, by Kupffer cells. We have shown that Kupffer cells engage in hepatic chemokine expression in response to brain injury^9^. Indeed, the selective depletion of these cells results in inhibition of leukocyte recruitment to the injured brain^34^, suggesting the EV signal from the brain is transduced by Kupffer cells into the traditional APR and resultant behaviour known to be associated with CNS injury.

The results described here (both *in vitro-*derived and *in vivo-*derived EVs) show that, not only are EVs able to initiate an APR, but they are also able to activate the sickness behaviours associated with injury or infection. We have previously shown that the circulating and hepatic expression of TNF-α is central to the sickness behaviours induced by the inflammatory lesions in the brain^5^. Etanercept, which is unable to cross the intact BBB, is able to block the sickness behaviours induced by the central injection of IL-1β. Therefore, it seems likely that the EV-induced hepatic TNF expression has the potential to induce the sickness behaviours observed in the present study.

## Conclusion

This study clearly highlights the capacity of EVs to alter cellular functions in distant organs. Overall, these data demonstrate an important novel mechanism of communication between the CNS and the liver, and provides a potential tool for future intervention. Target discovery, both on the surface and within EVs, is likely to provide essential evidence for the manipulation of the APR to CNS injury. By being able to intervene rapidly after a stroke or a traumatic brain injury, for example, and reduce the subsequent inflammation, we may significantly improve clinical outcomes and provide crucial extra time for neurological recovery.

## Materials and Methods

### Animals

Adult male Wistar rats were obtained from Harlan and housed under a standard 12-hour light/dark cycle. Animals were provided with food and water *ad libitum*. All procedures were carried out in accordance with the UK Animals (Scientific Procedures) Act, 1986 and licenced protocols were approved by local committees (LERP and ACER, University of Oxford). Animals were anaesthetized in a 2% isoflurane/oxygen mix (2 L/min) and placed in a stereotactic frame (Stoetling Co., USA) under maintenance anaesthesia (1.5%).

### Stereotaxic Surgery

All surgical procedures were performed under an operating microscope (Wild M650, Leica, Milton Keynes, UK). Recombinant rat interleukin-1β (rrIL-1β; 10ng/µl) (NIBSC, UK) was synthesized and stored in PBS until use^43^. Stereotaxic surgery was performed as described previously^44^. Briefly, anaesthetised rats were held in a stereotaxic frame, a burr hole was drilled in the skull and 1 µl of rrIL-1β was unilaterally microinjected into the left striatum through a finely-drawn glass microcapillary needle (tip < 50 µm) over a period of 5 minutes. The stereotaxic co-ordinates were -1.0 mm A/P, -3.0 mm M/L, and -4.0 mm D/V relative to Bregma. The dose of IL-1β in this study was chosen on the basis of our previous experiments and from those of others that have provoked a febrile response and behavioural depression^36^. IL-1β was dissolved in endotoxin-free saline (vehicle) and we have shown that the injected IL-1β does not make its way into the circulation^45^. During the surgical procedures, body temperature was maintained on a heated blanket throughout the period of anaesthesia, and the animals were allowed to recover in a heated chamber.

### Clodronate Treatment

Animals received either clodronate-filled liposomes or empty liposomes as an i.v. injection 24 hours prior to receiving either EVs from IL-1β-stimulated GP-8 cells or denatured EVs. EVs were denatured by boiling at 95 °C for 10 minutes.

### Nanoparticle Tracking Analysis (NTA)

All analyses were performed on a Nanosight NS500 NTA instrument (Malvern Instruments, Amesbury, UK) equipped with a 405 nm violet laser and a CMOS camera (Hamamatsu Photonics, Japan) as previously described^46^. The system uses a finely focused laser beam that is introduced to the sample through a glass prism. The beam refracts at a low angle as it enters the sample, resulting in a thin beam of laser light that illuminates particles through the sample. Particles resident within the beam are visualized using a conventional optical microscope, fitted with a video camera, aligned normally to the beam axis, which collects light scattered from all particles in the field of view. The sample chamber is 500 µm deep, but the beam depth is around 20 µm at the point of analysis, matching with the depth of focus of the imaging optics. A video of typically 60 seconds duration is taken, with a frame rate of 30 frames per second, and particle movement is analyzed by NTA software (NanoSight Ltd.). The operator was blinded to the identity of the samples. The samples were diluted to give a concentration of between 2 × 10^8^ and 10 × 10^8^. All samples were analysed using camera level 12. All videos were processed using NTA 2.3 build 17 software with threshold, minimum particle size and blur set to automatic. Five videos of 30 seconds were analysed using the following script: PUMPLOAD, REPEATSTART, DELAY 5, CAPTURE 30, PRIME, REPEAT 5. A suspension containing a known concentration of 100 nm diameter silica beads was analysed at the start and finish of each working day to ensure reproducibility of results. The NTA software is optimized to first identify and then track each particle on a frame-by-frame basis, and its Brownian movement tracked and measured frame to frame. The velocity of particle movement is used to calculate particle size by applying the two-dimensional Stokes-Einstein equation: $$ < x,y{ > }^{2}=\frac{{K}_{B}{T}_{t(s)}}{3\pi \eta {d}_{h}}$$ where $$ < x,y{ > }^{2}$$ is the mean squared displacement, K_B_ is Boltzmann’s constant, T is the temperature of the solvent in Kelvin, t_(s)_ is the sampling time (i.e., 1/30 fpsec = 33 msec), η is the viscosity, and d_h_ is the hydrodynamic diameter.

EV fractions were isolated from either platelet-free plasma or from cell culture supernatant centrifuged at 10,000 G for 30 minutes to separate out cellular debris. Extracellular vesicle fractions, where appropriate, were isolated from the supernatant of this spin using an ultracentrifuge at 120,000 G for 2 hours. Controls for each experiment were the supernatant fraction from this spin, derived from either platelet-free plasma or cell culture supernatant and described henceforth as EV-free supernatant.

### Extracellular Vesicle Transfer

EVs were isolated and measured in plasma from whole blood using NTA. For transfer experiments EVs were isolated and pooled from three donor animals and injected into one recipient animal (Fig. 2a). The pooled samples were quantified and each animal received 5 × 10^9^ particles in 0.5 ml of saline i.v.

### Cell Culture

Brain vascular endothelial cells (GP-8; ATCC) were cultured under standard conditions (95% air, 5% CO_2_, 37 °C) using DMEM (High Glucose – 4.5 g/L; Life Sciences) and 10% foetal calf serum with antibiotic and antimycotic. Cells were passaged using trypsin at 80–90% confluence and treated when confluent. IL-1β was used at a concentration of 10ng/ml in DMEM and cells were treated for 2–6 hours. In order to minimize ‘background’ EVs in serum and medium, both were filtered at 0.22 µm and ultracentrifuged for 18 hours at 120,000 G. Medium treated in this way did not affect the growth of the cells. For transfer of vesicles from *in vitro* cultures to a rat, a monolayer of a single T75 flask was used per animal yielding on average 1 × 10^10^ EVs per ml.

### Vesicle Labelling

EV fractions from cell culture experiments were used for *in vivo* tracking. EVs were isolated by ultracentrifugation as above. The final pellet was resuspended in EV-free PBS and combined 1:1 with PKH-67 (Sigma-Aldrich, UK) for 5 minutes in the dark to label the membrane of the EVs. The reaction was terminated using EV-free FCS and the labelled EVs were ultracentrifuged at 120,000 g for 2 hours before resuspension in EV-free 0.9% saline. For unlabelled, stimulation experiments the resuspended pellet was stored at 4 °C in PBS prior to use.

### Open Field Testing

Open field testing was carried out as described previously^5, 47^. The open field consisted of a rectangular wooden box 50 cm × 70 cm with 40 cm high walls, the rats were placed into a corner and allowed to explore for 3 minutes. Light levels were 350–400 Lux as used in previous experiments^48^. The movement of the animals was recorded on video and exploratory behaviour and rearing behaviour was analysed blind post-hoc. Rearing is considered to be an exploratory behaviour that is motivated by interest rather than a survival instinct to explore a novel environment. Distance travelled in an open field reflects the need to survey a new environment and will be reduced after the loss of rearing behaviour.

### Immunohistochemistry

Animals received an overdose of pentobarbital (i.p.) and were perfused with 0.9% saline until the liver was cleared of blood. Fresh liver was snap frozen using an isopentane bath on dry ice. 10 µm sections were cut on a cryostat (Leica CM1850) and post-fixed in formalin for 20 minutes before staining with CD11b (1:250, Abcam, Cambridge UK), and visualized using a biotinylated secondary antibody (Vectorlabs, UK) and streptavidin-linked Texas red (Vectorlabs) for fluorescent labelling. Slides were coverslipped using aqueous mounting medium containing DAPI (Vectorlabs). For neutrophil counting experiments, neutrophils were labelled with an in-house anti-neutrophil antibody (MBS-1) followed by a biotinylated secondary, developed using 3’3-diaminobenzidine and counterstained with haematoxylin. Counts were made using a 1 mm^2^ graticule in three areas on three separate sections and calculated as cells per mm^2^.

### Electron Microscopy

50 µl of purified EVs in 2% paraformaldehyde was applied to freshly glow-discharged carbon 300 mesh copper grids for 5 minutes. Samples were blotted with filter paper, passed through three droplets of distilled water (Milli-Q©), blotted again and then stained with 2% uranyl acetate for 10 seconds. Grids were blotted and air dried before being imaged in a FEI Tecnai 12 transmission electron microscope at 120 kV using a Gatan US1000 CCD camera.

### Quantitative PCR

RNA extraction and cDNA preparation were performed as previously described^49^. Samples were run using the Pfaffl method with a standard curve of mixed cDNA^50^. Primer and probe sets were designed by PrimerDesign Ltd. (Southampton, UK) with sequences as follows: TNF-F GCTCCCTCTCATCAGTTCCA; TNF-R CTCCTCTGCTTGGTGGTTTG; CXCL-1-F GCAGACAGTGGCAGGGATT; CXCL-1-R GTGGCTATGACTTCGGTTTGG. Samples were analyzed using a Roche Light Cycler 480® (Roche Diagnostics, Welwyn Garden City, UK) and SYBR green technology. Reaction efficiency was determined using the standard curve and the analysis was performed using a comparative cycle threshold method. Results were relative to the expression of the house keeping gene glyceraldehyde phosphate dehydrogenase (GAPDH) and all data were normalized to the corresponding saline group.

### IL-1β ELISA

To examine the distribution of IL-1β after injection, animals were injected with human recombinant IL-1β as previously described to enable the endogenous production to be distinguished from the exogenously administered cytokine. Recombinant human IL-1β is known to bind to rat IL-1R1 with high affinity^51^. After 2 hours blood was removed by cardiac puncture under terminal anesthesia; the brains were rapidly removed, sliced into 3-mm sections and both the injected and non-injected striata were punched out, snap-frozen in liquid nitrogen and stored at -80 °C for subsequent extraction and quantification for IL-1β by ELISAs specific for human or rat IL-1b (R&D Systems; UK). For optimal extraction of proteins, the tissue was homogenised on ice in phosphate buffer containing protease inhibitors [amino-n-caproic acid (100 mM), Na + EDTA (10 mM), benzamidine HCl (5 mM), AEBSF (0.2 mM)] at pH 7.4. Homogenates were centrifuged (12,000 g) for 10 min at 4 °C, and the supernatants were removed and assayed in duplicate immediately, along with calibrated cytokine standards. The sensitivities of these ELISA kits were all equal to or better than 15.6 pg/ml. Over 90% of a known amount of recombinant IL-1β standard added into normal brain homogenate or plasma (spike) was detected by this assay (recovery), indicating that neither known (endogenous antagonists) nor unknown biological factors interfere with binding.

### Data analysis

One and two-way ANOVA analysis and Student's t-tests were applied as necessary. Analysis was performed using GraphPad Prism 5 and SPSS. Data are presented as mean ± SEM.

## Electronic supplementary material

Supplementary Information

IL-1 NTA Video

PBS NTA Video

Supernatant NTA Video

Vehicle NTA Video
