# Supplementary material for: Circulating endothelial cell-derived extracellular vesicles mediate the acute phase response and sickness behaviour associated with CNS inflammation
Source: Sci Rep. 2017 Aug 29;7:9574. doi: 10.1038/s41598-017-09710-3 (PMC5575066; doi:10.1038/s41598-017-09710-3)
Supplement: Supplementary file 1 — Supplementary Information [file 41598_2017_9710_MOESM1_ESM.pdf]

Supplementary Information

Circulating endothelial cell-derived extracellular vesicles mediate the acute phase response and sickness behavior associated with CNS inflammation

Yvonne Couch[a*], Naveed Akbar[b], Jay Roodselaar[c] Matthew C. Evans[c], Chris Gardiner[d], Ian Sargent[e], Ignacio A. Romero[f], Adrian Bristow[g], Alastair M. Buchan[a], Norman Haughey[h], Daniel C. Anthony[c]

[a] Acute Stroke Programme, RDM-Investigative Medicine, University of Oxford, Oxford, UK
[b] Division of Cardiovascular Medicine, RDM, University of Oxford, Oxford, UK
[c] Pharmacology, University of Oxford, Oxford, UK.
[d] Haemostasis Research Unit, UCL, London, UK
[e] Department of Obstetrics and Gynaecology, University of Oxford, Oxford, UK.
[f] Department of Life, Health and Chemical Sciences, The Open University, Milton Keynes, UK
[g] NIBSC, South Mimms, Potters Bar, UK
[h] Department of Neurology and Psychiatry, Johns Hopkins University, Baltimore, Maryland, USA

*Corresponding author:

Dr. Yvonne Couch
Acute Stroke Programme
RDM Investigative Medicine
University of Oxford
John Radcliffe Hospital, OX3 9DU
Tel: (44) 01865 222049
email: yvonne.couch@rdm.ox.ac.uk

Key words: extracellular vesicles, inflammation, sickness behavior, liver

Supplementary

Figure Legends

Figure S1. IL-1β protein measured in rat brain homogenate, plasma or EV fractions by ELISA against (a) human IL-1β or (b) rat IL-1β. Values are expressed as pg/ml IL-1β. n.d. = not detectable. Data are mean ±SEM (n=3).

Figure S2. Western blot analysis of EVs isolated from the blood of sham and IL-1β-injected animals. (a) CD31 and CD41 (platelet marker) expression as a ratio of Tsg101 expression in the mixed vesicle sample. Note that while the platelet marker (CD41) increases in the IL-1β -challenged animals compared to controls, the increase is significantly greater for the CD31-positive EV population. The dissociation between CD31, which is also expressed in large EVs from platelets, and CD41expression confirms the enrichment of endothelial cell derived EVs. (b) Photographs of the immunostained blots of CD31, CD41, ALIX and TSG101 EV samples isolated from the plasma of control (saline) and IL-1β injected animals. (c) Electron micrographs of the isolated EVs fraction and EV-free plasma. Data are mean +/-SD (n=3); *** P=0.0007 CD31 compared to CD41 for IL-1β EVs, ++++ P=<0.0001 & +++ P=0.0002 Sham compared to IL-1β.

Figure S3. Lung and spleen neutrophil numbers in animals receiving EVs isolated from GP-8 supernatant. Total neutrophil numbers were counted in fixed tissue sections from (a) lung and (b) spleen). Data are mean ±SEM (n=4).

Figure S4. Fluorescence imaging of EVs. PHK-67 (green) labelled EVs were found in the spleen, lung, but not heart. However, compared to Liver (figure 3) the number of PHK-67 labelled EVs was far less prominent. Nuclei are labelled with DAPI (blue).

Supplementary Videos. NanoSight Tracking Analysis videos of blank PBS, EVs isolated from the plasma of animals after an intracranial injection of either saline or IL-1β and the supernatant/EV-free fraction from the EV isolation process. It should be noted that EV yield in the supernatant/EV-free

fraction was <10^6/ml and particles were an average size of <100nm and as such are likely to be small liposomes, rather than exosomes or microvesicles.

a

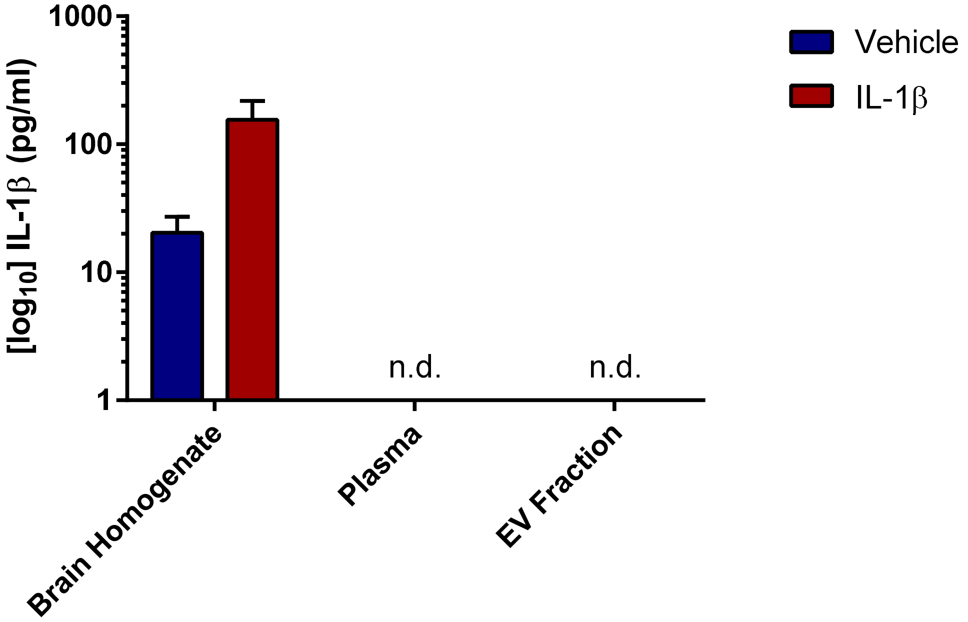

[log10] IL-1β (pg/ml)
1000
100
10
1
n.d.
n.d.
Brain Homogenate
Plasma
EV Fraction
Vehicle
IL-1β

b

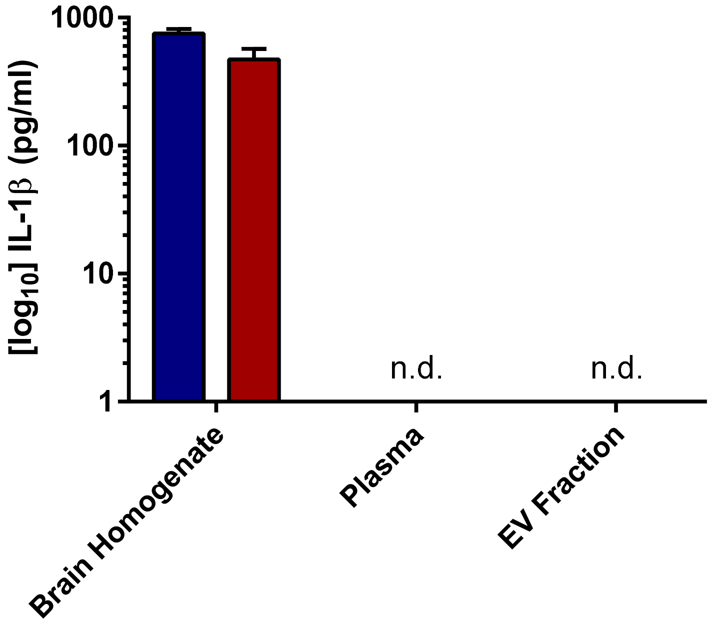

[log10] IL-1β (pg/ml)
1000
100
10
1
n.d.
n.d.
Brain Homogenate
Plasma
EV Fraction

a

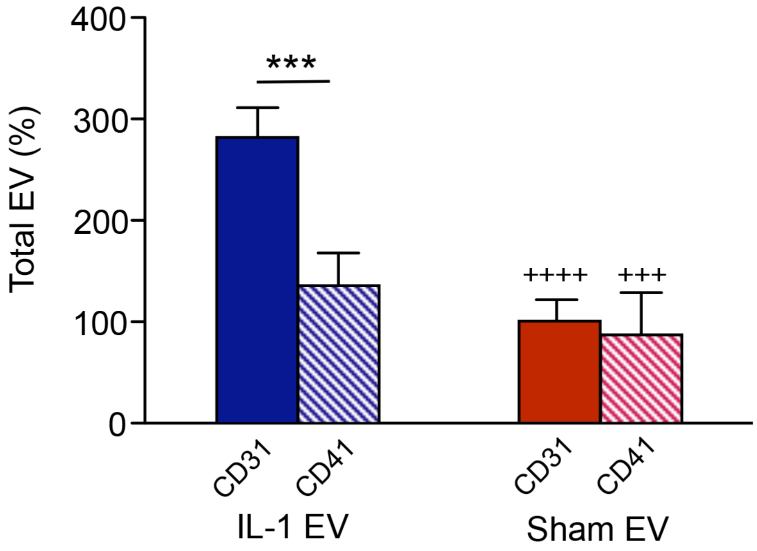

400
300
200
100
0
Total EV (%)
***
++++
+++
CD31
CD41
CD31
CD41
IL-1 EV
Sham EV

b

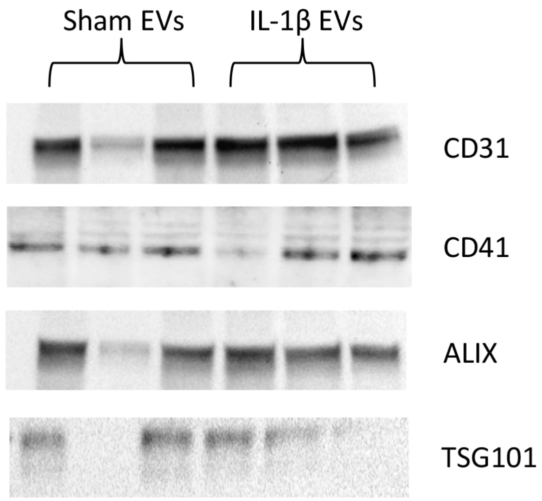

Sham EVs
IL-1β EVs
CD31
CD41
ALIX
TSG101

c

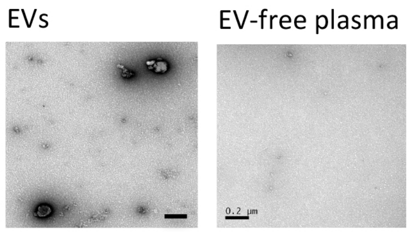

EVs
EV-free plasma
0.2 µm

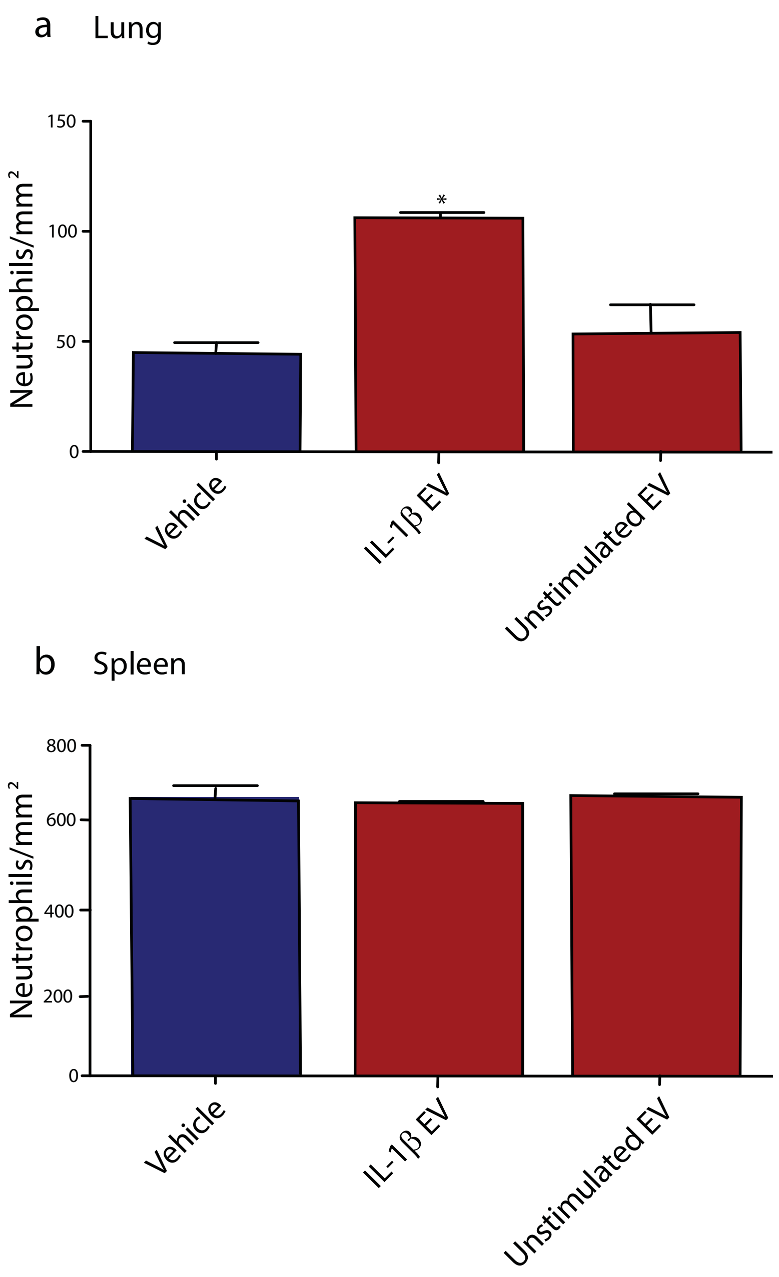
a
Lung
Neutrophils/mm²
150
100
50
0
*
Vehicle
IL-1β EV
Unstimulated EV
b
Spleen
Neutrophils/mm²
800
600
400
200
0
Vehicle
IL-1β EV
Unstimulated EV

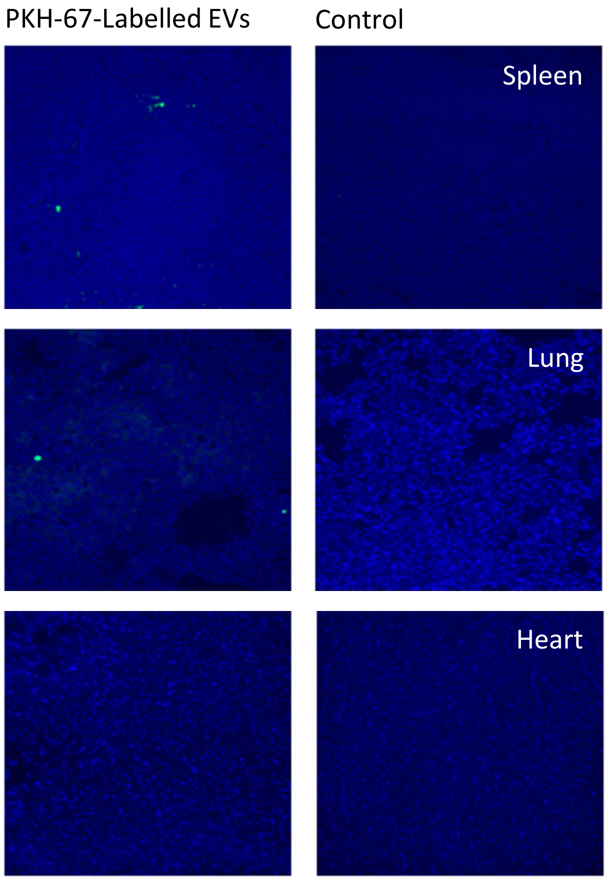
PKH-67-Labelled EVs
Control
Spleen
Lung
Heart
